# Changes in clinical symptoms and functional disability in patients with coexisting patellofemoral and tibiofemoral osteoarthritis: a 1-year prospective cohort study

**DOI:** 10.1186/s12891-017-1486-4

**Published:** 2017-03-24

**Authors:** Hirotaka Iijima, Naoto Fukutani, Takuya Isho, Yuko Yamamoto, Masakazu Hiraoka, Kazuyuki Miyanobu, Masashi Jinnouchi, Eishi Kaneda, Tomoki Aoyama, Hiroshi Kuroki, Shuichi Matsuda

**Affiliations:** 1 Department of Physical Therapy, Human Health Sciences, Graduate School of Medicine, Kyoto University, Kyoto, Japan; 2 Japan Society for the Promotion of Science, Tokyo, Japan; 3 Rehabilitation Center, Fujioka General Hospital, Gunma, Japan; 4 Nozomi Orthopaedic Clinic, Hiroshima, Japan; 5 Department of Orthopaedic Surgery, Graduate School of Medicine, Kyoto University, Kyoto, Japan

**Keywords:** Patellofemoral osteoarthritis, Tibiofemoral osteoarthritis, Knee pain, Disability

## Abstract

**Background:**

This 1-year prospective cohort study aimed to compare the changes in clinical symptoms and functional disability between patients with coexisting patellofemoral (PF) and tibiofemoral (TF) osteoarthritis (OA) and those with isolated TFOA.

**Methods:**

Seventy-two patients with medial knee OA were enrolled. Knee pain and functional disability were assessed at baseline and at 1-year follow-up using the Japanese Knee Osteoarthritis Measure (JKOM) and a visual analog scale (VAS). We performed two-way analysis of covariance for the clinical outcome variables to examine, time (baseline and follow-up), group (coexisting PFOA and isolated TFOA), and time-group interaction effects. Furthermore, we conducted post-hoc exploratory analysis to address the possibility that dividing patients according to location of PFOA (i.e., isolated lateral, isolated medial, and mixed [bilateral]) may identify a distinct subgroup with different changes in clinical outcomes at 1-year follow-up.

**Results:**

We detected group effects only in scores of the JKOM pain subscale (*P* = 0.012) and VAS (*P* = 0.033), adjusted for age, sex, and body mass index. Patients with coexisting PFOA have stable moderate level knee pain and functional disability throughout the year which is significantly worse than that in those with isolated TFOA. Post-hoc subgroup analysis demonstrated that change of knee pain likely varied with location of PFOA. Patients with isolated lateral PFOA had mild/moderate level knee pain, and their VAS scores were likely to improve, whereas those with mixed PFOA exhibited stable to worsening moderate/severe knee pain.

**Conclusions:**

Although we did not detect differences in changes in clinical symptoms and functional disability between patients with coexisting PFOA and those with isolated TFOA, our findings indicate that patients with coexisting PFOA had worse clinical symptoms and functional disability than those with isolated TFOA. The results of the exploratory analysis suggested that patients with coexisting PFOA might have heterogeneous clinical outcomes, and presence of mixed PFOA might be an indicator of severe clinical knee OA.

**Electronic supplementary material:**

The online version of this article (doi:10.1186/s12891-017-1486-4) contains supplementary material, which is available to authorized users.

## Background

Osteoarthritis (OA) of the knee is a common chronic and degenerative disease and a major cause of knee pain and functional disability worldwide [1]. Generally, knee OA is considered a progressive condition that requires total joint arthroplasty. However, several studies demonstrated that, while pain and functional disability generally worsened over time in patients with knee OA, individual outcomes were heterogeneous, with worsening in some patients and improvement in others [2–5]. Thus, there is a possibility that the pool of patients with knee OA comprises a number of subgroups with distinct trajectories of pain and functional disability, not all of which are progressive. Since identifying these homogenous subgroups would provide information about clinical prognosis and facilitate targeted treatment, this topic has become a recent focus of epidemiologic and clinical studies of knee OA [6, 7].

Although patellofemoral (PF) OA remains an under recognized type of knee OA, patients with PFOA have been recently considered a subgroup different from patients with tibiofemoral (TF) OA [8]. Duncan et al. demonstrated that the prevalence of mixed PFOA and TFOA in older adults with painful knees is much higher (40%) than that of TFOA (4%) or PFOA (24%) alone [9]. Since the PF joint contributes more to the symptoms of knee OA than the TF joint dose [10], presence of PF joint disease can adversely affect physical activity, participation in social life, and quality of life. Indeed, recent studies demonstrated that patients with coexisting PFOA and TFOA were more likely to have pain and functional disability as well as knee-specific impairments such as quadriceps weakness and restricted range of motion of the knee joint than those with isolated TFOA did [11–14]. Importantly, increased knee pain and knee-specific impairments are potential risk factors for disease progression [15], worsening knee pain [16], and activity limitation [4, 17]. Furthermore, patients with coexisting PFOA are known to have altered gait biomechanics with more frequent single-leg stance external knee flexion moments [14], which elevates compressive stresses of the PF [18] and TF [19] joints and increases the risk of progression of clinical and radiographic OA.

These studies suggest that patients with coexisting PFOA may have a natural course of the disease different from that in patients with isolated TFOA and may exhibit worsening clinical symptoms and functional disability over time. However, previous studies that investigated coexisting PFOA had a cross-sectional design [12, 13]. A longitudinal cohort study would provide important insights into the clinical prognosis and treatment options. Additionally, previous studies that attempted to identify distinct OA subgroups did not consider the PF joint [4, 20, 21], although the trajectory of PF joint disease may differ from that of TF joint disease [22]. Given that interventions targeting the PF joint are recommended for patients with PF joint disease [23–25], such information may help to design an optimal treatment based on the involvement of different compartments. Therefore, this study aimed to compare the changes in clinical symptoms and functional disability differences between patients with coexisting PFOA and isolated TFOA. It was hypothesized that, (i) compared to patients with isolated TFOA, those with coexisting PFOA would have worsening clinical symptoms and functional disability at 1-year follow-up, and (ii) dividing patients according to location (i.e., isolated lateral, isolated medial, and mixed) of PFOA could identify a distinct subgroup with different clinical outcomes at 1-year follow-up.

## Methods

### Study design and patients

As described in the previous study [12], 143 patients with medial knee OA were recruited from a community orthopedics clinic in February, 2014. Patients were identified through the medical record system, and were consecutively recruited from the community orthopedics clinic in Hiroshima, which is located in a rural, mountainous community. We distributed an advertisement requesting patients who were visiting the clinics for conservative treatment of knee OA. All recruited patients had a history of pain in one or both knees. The patients were followed up for 12 months. Inclusion criteria were (i) age ≥50 years; (ii) radiographic OA of one or both knees with a Kellgren/Lawrence [K/L] grade [26] ≥ 2, primarily in the medial tibiofemoral compartment, as evaluated using anteroposterior weight-bearing radiographs of the TF joint; and (iii) ability to walk independently on a flat surface, without walking aids. Patients with bilateral knee OA were not considered separately from unilateral cases. The exclusion criteria were (i) a history of knee surgery, (ii) inflammatory arthritis, (iii) periarticular fracture, (iv) presence of neurological diseases, or (v) lateral compartment knee OA. Lateral knee OA was defined as a knee having a K/L grade ≥2, along with lateral joint space narrowing (JSN) > medial JSN, and lateral osteophytes > medial osteophytes, using an Osteoarthritis Research Society International (OARSI) atlas [27] according to previously described methods [28, 29]. The Ethical Committee of Kyoto University approved this study (approval number: E1923), and written informed consent was obtained from all participants before enrollment both at baseline and 1-year follow-up.

### Baseline radiographic evaluation of tibiofemoral and patellofemoral joints

Methods for baseline radiographic evaluation of severity of OA of the TF and PF joints determination were described in detail elsewhere [12]. In brief, the radiographic severity for the TF joint was assessed using the K/L grading system on the anteroposterior short film view in the weight-bearing position by an experienced examiner (TA). Similarly, radiographic severity for the PF joint was evaluated using the K/L grading system adapted to the lateral and medial facets of the PF joint by a single trained examiner (HI). We have previously reported excellent intra-rater reliability for such radiographic evaluation, with a Kappa coefficient of 0.90 (TF) and 0.80 (PF) [12]. Patellar alignments and trochlear morphology were evaluated from the skyline view by a single trained examiner (HI) who evaluated lateral displacement, tilting angle of the patella, and sulcus angle [12]. Details regarding the measurement for the patellar alignments were described recently [12]. The intrarater reliabilities were excellent for the lateral displacement (intraclass correlation coefficient [ICC] = 0.91, 95% confidence interval [CI] = 0.89–0.93) and tilting angle of the patella (ICC = 0.96, 95% CI = 0.95–0.97).

### Primary outcomes

Severity of self-reported knee pain was evaluated individually at baseline and 1-year follow-up, using a visual analog scale (VAS) and the “pain and stiffness” subcategory of the Japanese Knee Osteoarthritis Measure (JKOM) in person-specific assessments. The VAS score was interpreted as no pain (VAS ≤ 10 mm), mild pain (10 mm < VAS ≤ 30 mm), moderate pain (30 mm < VAS ≤ 60 mm), or severe pain (VAS > 60 mm) based on previous studies [30, 31]. The JKOM is a patient-based, self-administered scoring system that includes four subcategories assessing “pain and stiffness” (8 questions, 0–32 points), “activities of daily living” (10 questions, 0–40 points), “participation in social activities” (5 questions, 0–20 points), and “general health conditions” (2 questions, 0–8 points), with 100 points set as the maximum score. For each subscale, a higher score indicates a worse condition (on a 0–4 Likert scale, 0 indicates no pain or difficulty while 4 represents extreme pain or difficulty). The concurrent and construct validities of the JKOM were established by comparing with the Western Ontario and McMaster Universities Arthritis Index and the Medical Outcomes Study 36-item Short-Form Health Survey [32].

### Secondary outcomes

Secondary outcomes included the other JKOM subcategories (“activities of daily living,” “participation in social activities,” and “general health conditions”) evaluated at both baseline and 1-year follow-up. The JKOM subcategory “activities of daily living” is a self-reported physical functional assessment reflecting daily activities such as stair use, bending, standing up from a sitting position, walking, shopping, taking off socks, and performing light and heavy household duties.

### Measurement of covariates

Data on age, sex, and height were self-reported by the patients. Weight was measured on a scale, with the participants wearing their clothes but not their shoes. Body mass index (BMI) was calculated by dividing the weight by the square of height.

### Statistical analysis

To minimize bias produced by similarities between the right and left knees of the same patient [33], only one knee per patient was analyzed (“index knee”), and a database was created with one observation per patient. The index knee was defined as the more painful knee, currently or in the past. If a patient reported equal pain in both knees, the index knee was selected randomly. All continuous data were assessed for following a Gaussian distribution using the Shapiro–Wilk normality test, and for homoscedasticity using the F-test. Baseline demographic characteristics, including radiographic OA severity, were then compared between patients with coexisting TFOA and PFOA and isolated TFOA, using the Student’s *t*-test for parametric continuous variables, Mann-Whitney *U* test for nonparametric continuous variables, or chi-square/Fisher’s exact tests for dichotomous/categorical variables. Next, we performed two-way repeated analysis of covariance (ANCOVA) for clinical outcome variables (JKOM and VAS scores) to assess the interaction between time (baseline and 1-year follow-up) and group (coexisting PFOA and isolated TFOA) adjusted for age (continuous), sex, and BMI (continuous) and further adjusted for TF joint K/L grade (continuous). As age, sex, BMI, and TF joint K/L grade were likely to affect the clinical outcome of OA [2, 20] and not on the causal pathway, we included these confounders in the model.

To verify the possibility that dividing patients according to location of PFOA may identify a distinct subgroup with different trajectories of clinical outcomes at 1-year follow-up, we performed explorative subgroup analysis of the relationships between location of PFOA (i.e., isolated lateral, isolated medial, and mixed [bilateral]) and outcome variables by using subsamples of patients with coexisting PFOA. We performed a two-way repeated ANCOVA for the clinical outcome variables to assess the interaction between time (baseline and 1-year follow-up) and group (isolated lateral, isolated medial, and mixed PFOA) adjusted for age, sex, BMI, and further adjusted for TF joint K/L grade, with post-hoc pairwise comparisons. Furthermore, we calculated the percentage of patients having unacceptable symptoms representing a clinically relevant treatment target, defined as a VAS score above 30 mm [31, 34, 35], and compared these percentages between the groups using the chi-square test. In the sensitivity analyses, the type I error rate was not adjusted for multiple pairwise comparisons as the analysis was exploratory in nature. All data analyses were performed with JMP 11 (SAS Institute, Cary, NC, USA) or R (R Foundation for Statistical Computing, Vienna, Austria). *P*-values <0.05 were considered to indicate statistical significance.

## Results

Our final sample included 72 patients/knees (response rate: 50.3%) with a K/L grade ≥2 primarily for the medial compartment. Patients who failed to complete the study were unable to be contacted or declined to be followed for non-specific reasons. Baseline characteristics were compared between completers and non-completers (Table 1), and no significant differences were found between the two groups in demographic characteristics, OA disease severity, location of PFOA, patellar alignment, knee pain intensity, and functional disability. Of the 72 patients who completed the study (age, 56–90 years; 73.6% female), 45 (62.5%) had PFOA with a K/L grade ≥2 of either the lateral or the medial PF joint of their index knee. Table 2 shows the baseline characteristics of patients with coexisting PFOA and TFOA (n = 45) and those with isolated TFOA (without PFOA, n = 27). As mentioned in the recently published paper [12], compared to patients with isolated TFOA, those with coexisting PFOA tended to be older and had a significantly higher BMI and a more advanced disease of the TF joint. Moreover, patients with coexisting PFOA and TFOA had significantly greater lateral displacement and lower tilting angle of the patella (i.e., less lateral rotational position of the patella) than those with isolated TFOA. Most patients with coexisting PFOA had either lateral (18 [40.0%]) or mixed (23 [51.1%]) PFOA, with patients with lateral PFOA displaying milder disease of the TF and PF joints compared to those with mixed PFOA (see Additional file 1: Table S1).

**Table 1:** Comparison of baseline characteristics between patients with completer and those with non-completer^a^

Variables	Completer (*n* = 72)	Non-completer (*n* = 71)	*P*-value**
Age, years	73.9 ± 8.12	73.5 ± 7.17	0.754
Women, no. (%)	53 (73.6)	55 (77.5)	0.945
Height, meters	1.55 ± 0.08	1.54 ± 0.06	0.862
Weight, kg	58.2 ± 10.3	59.4 ± 10.4	0.471
Body mass index, kg/m2	23.8 ± 4.66	24.9 ± 4.26	0.389
Tibiofemoral joint K/L grade, no. (%)			0.901
Grade 2	48 (66.7)	46 (64.8)	
Grade 3	14 (19.4)	16 (22.5)	
Grade 4	10 (13.9)	9 (12.7)	
Patellofemoral joint K/L grade, no. (%)			0.278
Grade 0	2 (2.8)	0 (0.0)	
Grade 1	25 (34.7)	18 (25.4)	
Grade 2	30 (41.7)	32 (45.1)	
Grade 3	9 (12.5)	16 (22.5)	
Grade 4	6 (8.3)	5 (7.0)	
Location of patellofemoral osteoarthritis, no. (%)			0.522
Medial facet	4 (5.6)	2 (2.8)	
Lateral facet	18 (25.0)	25 (35.2)	
Mixed (medial and lateral) facet	23 (31.9)	26 (36.6)	
Patellar alignment and trochlear morphology			
Lateral displacement, %	8.64 ± 5.97	10.2 ± 5.54	0.077
Tilting angle, degrees^b^	5.43 ± 4.15	6.47 ± 4.59	0.114
Sulcus angle, degrees	133.9 ± 6.61	132.7 ± 5.25	0.237
JKOM			
Pain and stiffness (0–32 points)	8.82 ± 6.05	9.48 ± 5.69	0.444
Activities of daily living (0–40 points)	7.97 ± 7.40	8.45 ± 6.61	0.394
Participation in social activities (0–20 points)	4.06 ± 4.21	3.63 ± 3.85	0.548
General health conditions (0–8 points)	2.99 ± 1.65	2.96 ± 1.39	0.821
Total score (0–100 points)	23.8 ± 16.3	24.5 ± 14.6	0.586
VAS score for knee pain, mm	3.56 ± 2.90	3.71 ± 2.74	0.617

*K*/*L* grade Kellgren/Lawrence grade, *JKOM* Japanese Knee Osteoarthritis Measure, *VAS* Visual analog scale

**Based on unadjusted analysis (Student’s *t*-test [age, weight, and sulcus angle], Mann-Whitney *U* test [height, body mass index, lateral displacement, tilting angle, JKOM score, and VAS score for knee pain], or chi-square/Fisher's exact tests [sex, tibiofemoral and patellofemoral joint K/L grades, and location of patellofemoral osteoarthritis]) of the completer and non-completer groups

^a^Except where otherwise indicated, values are mean ± SD

^b^A positive value for tilting angle indicates patellar tilt toward the lateral side and a negative value to the medial side

**Table 2:** Comparison of characteristics between patients with coexisting PFOA and those with isolated TFOA^a^

Variables	TFOA + PFOA (*n* = 45)	Isolated TFOA (*n* = 27)	*P*-value^**^
Age, years	75.2 ± 8.86	71.6 ± 6.21	0.064
Women, no. (%)	33 (73.3)	20 (74.0)	0.945
Height, meters	1.54 ± 0.08	1.57 ± 0.08	0.120
Weight, kg	59.6 ± 10.2	55.7 ± 10.2	0.125
Body mass index, kg/m2	25.1 ± 3.76	21.7 ± 5.29	**0.002**
Tibiofemoral joint K/L grade, no. (%)			**0.001**
Grade 2	23 (51.1)	25 (92.6)	
Grade 3	13 (28.9)	1 (3.7)	
Grade 4	9 (20.0)	1 (3.7)	
Patellofemoral joint K/L grade, no. (%)			–
Grade 0	0 (0)	2 (7.4)	
Grade 1	0 (0)	25 (92.6)	
Grade 2	30 (66.7)	0 (0)	
Grade 3	9 (20.0)	0 (0)	
Grade 4	6 (13.3)	0 (0)	
Location of patellofemoral osteoarthritis, no. (%)			–
Medial facet	4 (8.9)	–	
Lateral facet	18 (40.0)	–	
Mixed (medial and lateral) facet	23 (51.1)	–	
Patellar alignment and trochlear morphology			
Lateral displacement, %	10.4 ± 5.87	5.73 ± 5.01	**<0.001**
Tilting angle, degrees^b^	4.52 ± 4.27	6.95 ± 3.54	**0.009**
Sulcus angle, degrees	133.6 ± 6.49	134.4 ± 6.89	0.602

*TFOA* Tibiofemoral osteoarthritis, *PFOA* Patellofemoral osteoarthritis, *K/L grade* Kellgren/Lawrence grade

*P*-values indicating statistically significant differences (<0.05) are displayed in bold

^**^Based on unadjusted analysis (Student’s *t*-test [age, weight, and sulcus angle], Mann-Whitney *U* test [height, body mass index, lateral displacement, and tilting angle], or chi-square/Fisher's exact tests [sex, tibiofemoral and patellofemoral joint K/L grades, and location of patellofemoral osteoarthritis]) of the TFOA + PFOA and isolated TFOA groups

^a^Except where otherwise indicated, values are mean ± SD

^b^A positive value for tilting angle indicates patellar tilt toward the lateral side and a negative value to the medial side

### Clinical symptoms and functional disability and their time dependence

Two-way repeated ANCOVA revealed no significant time-group interactions at any outcome variables, and only the group main effect was confirmed for all the outcome variables adjusted for age, sex, and BMI (Table 3). Further adjustment for TF joint K/L grade did not substantially change this result (data not shown). Patients with coexisting PFOA had worse clinical symptoms throughout the year than did those with isolated TFOA as reflected by higher scores in the “pain and stiffness” subcategory and on the VAS pain score. Thus, moderate pain (30 mm < VAS ≤ 60 mm) persisted in patients with coexisting PFOA, although the standard deviations of the changes in VAS pain score were large (10 [22.2%] and 18 [40.0%] patients reported improving and worsening categorized VAS scores, respectively). Additionally, patients with coexisting PFOA had higher scores in the “activities of daily living” subcategory (i.e., had more difficulty in daily living) throughout the year.

**Table 3:** Changes in the JKOM and VAS scores after 1 year of follow-up

Variables	TFOA + PFOA (*n* = 45)			Isolated TFOA (*n* = 27)			Two-way ANCOVA^a^		
	Baseline	1-year follow-up	Mean change	Baseline	1-year follow-up	Mean change	Time^b^	Group^c^	Interaction
	Mean ± SD	Mean ± SD	Mean (95% CI)	Mean ± SD	Mean ± SD	Mean (95% CI)	Adjusted *P*-value		
JKOM									
Pain and stiffness (0–32 points)	10.9 ± 6.14	11.3 ± 8.31	0.38 (-1.16, 1.92)	5.22 ± 3.72	4.85 ± 4.28	-0.37 (-1.72, 0.98)	0.996	**<0.001**	0.738
Activities of daily living (0–40 points)	11.2 ± 8.93	12.1 ± 9.10	0.93 (-1.18, 3.04)	4.15 ± 3.73	5.19 ± 5.31	1.04 (-0.43, 2.51)	0.430	**<0.001**	0.965
Participation in social activities (0–20 points)	5.33 ± 4.64	4.33 ± 4.24	-1.00 (-2.47, 0.47)	2.00 ± 2.12	2.62 ± 3.68	0.62 (-0.50, 1.73)	0.778	**0.010**	0.237
General health conditions (0–8 points)	3.20 ± 1.84	3.33 ± 1.51	0.13 (-0.42, 0.68)	2.63 ± 1.21	2.48 ± 1.50	-0.15 (-0.58, 0.28)	0.978	**0.100**	0.601
Total score (0–100 points)	30.0 ± 17.5	30.1 ± 20.0	0.12 (-4.24, 4.48)	13.8 ± 7.93	15.3 ± 11.7	1.48 (-2.07, 5.03)	0.769	**<0.001**	0.803
VAS score for knee pain, mm	40.8 ± 27.2	43.1 ± 30.9	2.33 (-6.15, 10.8)	26.9 ± 30.2	20.0 ± 18.8	-6.89 (-18.3, 4.57)	0.633	**0.003**	0.334

*TFOA* Tibiofemoral osteoarthritis, *PFOA* Patellofemoral osteoarthritis, *ANCOVA* Analysis of covariance, *95% CI* 95% confidence interval, *JKOM* Japanese Knee Osteoarthritis Measure, *VAS* Visual analog scale

*P*-values indicating statistically significant differences (<0.05) are displayed in bold

^a^Adjusted *P*-values were calculated from the ANCOVA adjusted for age, sex, and body mass index

^b^Baseline vs. 1-year follow-up

^c^PFOA + TFOA vs. isolated TFOA

### Explorative subgroup analysis: time dependence of severity of clinical symptoms and functional disability, and possible dependence of pain trajectory on location of patellofemoral osteoarthritis

Explorative subgroup analysis stratified for the patients with coexisting PFOA showed that pain trajectory depended on the location of PFOA (Table 4). Two-way repeated ANCOVA revealed no significant time-group interactions, and only the group main effect was confirmed for the “pain and stiffness” subcategory (adjusted *P*-value = 0.012) and VAS score (adjusted *P*-value = 0.033). Nevertheless, clinical symptoms in patients with isolated lateral PFOA were likely to decrease during the 1-year follow-up (change in “pain and stiffness”: -1.89, 95% CI: -4.31, 0.53; change in VAS: -10.8; 95% CI: -21.5, -0.08). In contrast, clinical symptoms in patients with mixed PFOA were moderate/severe and stable or worsening throughout the year (change in “pain and stiffness”: 2.00, 95% CI: -2.71, 6.71; change in VAS: 9.17; 95% CI: -21.0, 39.3). Additionally, a multiple comparison revealed that “pain and stiffness” and VAS scores in patients with mixed PFOA were significantly higher than in those with isolated lateral PFOA when adjusted for age, sex, and BMI (adjusted *P*-values for post-hoc pairwise comparison: 0.021 and 0.022, respectively). Moreover, patients with mixed PFOA had consistently worse scores in the other JKOM subcategories and total JKOM score. When TF joint K/L grade was further adjusted for in the two-way repeated ANCOVA model, all the main group effects were attenuated (data not shown).

**Table 4:** Subgroup analysis of changes in the JKOM and VAS scores in patients with coexisting PFOA according to PFOA location

Variable	Isolated lateral (*n* = 18)			Isolated medial (*n* = 4)			Mixed (*n* = 23)				Two-way ANCOVA^a^		
	Baseline	1-year follow-up	Mean change	Baseline	1-year follow-up	Mean change	Baseline	1-year follow-up	Mean change		Time^b^	Group^c^	Interaction
	Mean ± SD	Mean ± SD	Mean (95% CI)	Mean ± SD	Mean ± SD	Mean (95% CI)	Mean ± SD	Mean ± SD	Mean (95% CI)		Adjusted *P*-value		
JKOM													
Pain and stiffness (0–32 points)	9.28 ± 6.17	7.39 ± 7.62	-1.89 (-4.31, 0.53)	12.5 ± 4.79	14.8 ± 9.11	2.50 (-2.40, 7.40)	12.2 ± 6.25	14.2 ± 7.64	2.00 (-2.71, 6.71)	^d^	0.654	**0.012**	0.426
Activities of daily living (0–40 points)	9.06 ± 7.68	9.11 ± 9.56	0.06 (-3.96, 4.07)	11.5 ± 8.58	15.3 ± 12.7	3.75 (-2.04, 9.54)	12.8 ± 9.69	14.1 ± 7.60	1.14 (-4.88, 7.17)		0.438	0.057	0.823
Participation in social activities (0–20 points)	5.06 ± 5.06	3.06 ± 4.40	-2.00 (-4.34, 0.34)	4.50 ± 2.65	6.75 ± 4.99	2.25 (-0.33, 4.83)	5.70 ± 4.69	4.91 ± 3.85	-0.78 (-5.88, 4.31)		0.883	0.531	0.444
General health conditions (0–8 points)	2.61 ± 1.82	2.72 ± 1.84	0.11 (-0.97, 1.20)	2.50 ± 0.58	3.75 ± 0.96	1.25 (0.02, 2.48)	3.78 ± 1.86	3.74 ± 1.14	-0.04 (-1.53, 1.45)	^d^	0.321	**0.016**	0.566
Total score (0–100 points)	25.2 ± 17.5	21.6 ± 21.3	-3.61 (-11.7, 4.47)	30.0 ± 14.8	39.3 ± 25.5	9.25 (-3.03, 21.5)	34.3 ± 17.6	36.0 ± 15.2	1.65 (-10.3, 13.6)	^d^	0.607	**0.013**	0.572
VAS score for knee pain, mm	38.9 ± 30.1	28.2 ± 30.2	-10.8 (-21.5, -0.08)	24.5 ± 15.9	46.5 ± 34.6	22.0 (0.64, 43.3)	45.1 ± 26.0	54.3 ± 26.8	9.17 (-21.0, 39.3)	^d^	0.385	**0.033**	0.167

*PFOA* Patellofemoral osteoarthritis, *ANCOVA* Analysis of covariance, *95% CI* 95% confidence interval, *JKOM* Japanese Knee Osteoarthritis Measure, *VAS* Visual analog scale

*P*-values indicating statistically significant differences (<0.05) are displayed in bold

^a^Adjusted *P*-values were calculated from the ANCOVA adjusted for age, sex, and body mass index

^b^Baseline vs. 1-year follow-up

^c^Isolated lateral vs. isolated medial vs. mixed

^d^Significantly different (*P* <0.05) from the isolated lateral group when adjusted for age, sex, and body mass index as a post-hoc test of ANCOVA

**Fig. 1:**
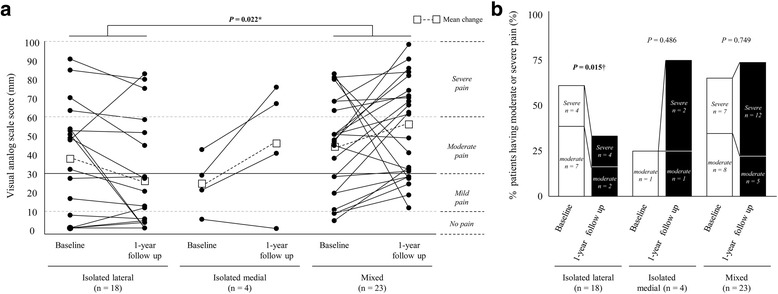
Changes in VAS in the subpopulation of patients with coexisting PFOA at 1 year follow-up. **a**, Trajectories of VAS scores. Categorized pain scale grades are also shown in the same panel (no, mild, moderate, and severe). The clinically important borderline between mild and moderate pain (VAS score = 30 mm) is displayed as a *solid horizontal line*. Other borderline values on the categorized pain scale are displayed as *dotted horizontal lines*. *White squares* connected with *dotted lines* represent the mean change in VAS score of each group. ^*^Significantly different (*P* < 0.05) after adjusting for age, sex, and body mass index in a post-hoc analysis of two-way repeated analysis of covariance (time effect: adjusted *P*-value = 0.385, group effect: adjusted *P*-value = 0.033, time-group interaction: adjusted *P*-value = 0.167, as shown in Table 4). **b**, Numbers and percentages of patients with moderate or more severe pain (i.e., moderate [30 mm < VAS ≤ 60 mm] and severe [VAS > 60 mm]) corresponding to a state with unacceptable symptoms and considered a clinically relevant treatment target [31, 34, 35]. Unadjusted *P*-values were calculated using the chi-square test. ^†^ *P*-values corresponding to significant differences are displayed in *bold*

Individual pain trajectories (Fig. 1a) revealed that 7 (38.9%) of the 18 patients with isolated lateral PFOA were transferred to a lower pain category, whereas none of the patients with isolated medial PFOA was transferred to a lower pain category and 3 (75%) of the 4 were transferred to an upper pain category. Of the 23 patients with mixed PFOA, 13 (56.5%) and 3 (13.0%) were transferred to an upper or lower pain category, respectively. Fig. 1b shows numbers and percentages of patients with moderate (i.e., 30 mm < VAS ≤ 60 mm) or severe knee pain (i.e., VAS > 60 mm) corresponding to a state with unacceptable symptoms and considered a clinically relevant treatment target [31, 34, 35]. The percentage of individuals having unacceptable symptoms significantly decreased in patients with isolated PFOA at 1-year follow-up (from 61.1% to 33.3%; unadjusted *P*-value = 0.015), but not in those with isolated medial (unadjusted *P*-value = 0.486) and mixed (unadjusted *P*-value = 0.749) PFOA. Importantly, more than 65% of patients with mixed PFOA had consistently unacceptable symptoms throughout the year (change: from 65.1% to 73.9%).

## Discussion

There is growing evidence that coexisting PFOA in patients with knee OA is common [9], and this mixed disease is associated with worse clinical symptoms and low quality of life [11, 12]. Although patients with coexisting PFOA are more likely to have potential risk factors for progression of clinical knee OA because of knee-specific impairments and altered gait biomechanics [12–14], little is known about the course of clinical symptoms and functional disability in such patients. The present study revealed that clinical symptoms and functional disability in patients with coexisting PFOA were persistently bad, but not worsening, throughout the year (Table 3). Additionally, the exploratory subgroup analysis demonstrated a possible dependence of pain trajectory on the location of PFOA within the coexisting PFOA subgroup. Patients with mixed PFOA exhibited stable or worsening moderate/severe clinical symptoms, whereas clinical symptoms were likely to improve in those with isolated lateral PFOA (Table 4 and Fig. 1).

Recently, Nicholls et al. characterized distinct trajectories of knee pain using latent class growth curve modeling analysis, showing that patients labeled “mild, non-progressive,” “moderate,” or “severe, non-improving” constituted stable subgroups of two independent large datasets [5]. Other studies also demonstrated that trajectories of pain severity and self-reported functional disability in patients with knee OA are often non-progressive over several years [20, 36]. Although we did not directly identify distinct subgroups using statistical methods such as latent class growth modeling [5] and latent class cluster analysis [21], patients with coexisting PFOA and TFOA in the present study likely correspond to the “severe, non-improving” class with consistently severe symptoms (Table 3). The presence of persistently bad but not worsening pain and functional disability throughout the year was unexpected. Our results may indicate that clinical symptoms and functional disability in patients with mixed TF and PF joints disease are not necessarily progressive despite the more frequent presence of risk factors of progression of clinical OA, such as quadriceps weakness [13, 14] and altered gait biomechanics [14]. Nevertheless, the longer duration of worse clinical symptoms and pain-related functional disability in patients with coexisting PFOA could lead to a further functional decline, limited participation in social activities, and decreased quality of life. A long-term prospective study with a large sample size, following the trajectory of the clinical symptoms and functional disability in patients with coexisting PFOA and TFOA, would be of particular interest in answering this question.

Previous longitudinal studies of clinical symptom progression suggested heterogeneous outcomes in patients with knee OA, with knee pain improving in only some patients [2–5]. In the present study, heterogeneous outcomes in terms of knee pain were confirmed in patients with coexisting PFOA: 10 (22.2%) and 18 (40.0%) of the 45 patients showed improvement and worsening on categorized VAS, respectively. To test the possibility that categorizing the patients according to location of PFOA may identify distinct subgroups with different trajectories of clinical outcomes at 1-year follow-up, we performed explorative subgroup analysis (Table 4 and Fig. 1) and found that patients with mixed PFOA had consistently severe clinical symptoms, which exceeded the unacceptable level (VAS > 30 mm) [34, 35] in more than 65% of the patients after 1 year, whereas knee pain in patients with isolated lateral PFOA was likely to decrease during this period. Stefanik et al. showed that, in the Multicenter OA study and Framingham OA cohort, magnetic resonance imaging (MRI)-detected full-thickness cartilage loss and bone marrow lesion of any size in mixed PF joints were more likely to be associated with knee pain than the same findings in isolated medial or lateral PF joints [37]. Given that adjusting for TF joint K/L grade reduced the significant difference in knee pain between patients with mixed PFOA and isolated lateral PFOA (data not shown), TF joint severity could also contribute to knee pain differences between the groups. Nevertheless, our findings suggest that the heterogeneity of clinical outcomes in patients with coexisting PFOA should be recognized, and mixed PF joint disease might indicate a subgroup with more severe clinical symptoms that are likely to exceed the unacceptable level.

Interestingly, the improvement in clinical symptoms in patients with isolated lateral PFOA may not be accompanied by significant functional improvement (Table 4). This may be a consequence of avoidance of activities because of anticipated pain and low vitality [38]. Therefore, pain reduction should not be considered equivalent to functional improvement, particularly that of performance-based physical function [39, 40].

Our study has several limitations to be noted. It is unknown whether the clinical symptoms and functional disability at the 1-year follow-up were affected by a bias due to missing values during this period. Therefore, follow-up losses need to be minimized to exactly characterize pain and functional changes in patients with coexisting PFOA. However, there were no significant differences between patients who completed the follow-up study and those who did not in baseline characteristics, which might indicate that data are missing at random. We confirmed that baseline knee pain intensity, which is a previously reported prognostic factor for worsening pain and disability [41], did not differ between completer and non-completer. Another important limitation is the small sample size, as the study might not have sufficient statistical power to detect a potential association between presence of PFOA and clinical outcome changes, particularly in patients with isolated medial PFOA. Nevertheless, the result from the explorative subgroup analysis demonstrated a potential dependence of pain trajectory on the location of PFOA in patients with coexisting PFOA; it is important to verify this finding in a larger study with a long-term follow-up. Our finding may help to set the foundation for a prospective cohort study with the aim of better understanding different pain and functional trajectories in patients with knee OA. Furthermore, the lack of patient information about pain medication and rehabilitation treatment may have restricted our analysis. These treatment effects might contribute to the dependence of pain trajectory on location of PFOA. Finally, we did not evaluate the location of knee pain within the knee joint, including the PF joint, although Stefanik et al. showed that self-reported location of the anterior knee pain is not highly specific in PF lesions detected with MRI [42]. As also mentioned in the recently published paper [12], altered patellar alignment was not a predictor of knee pain intensity in patients with coexisting PFOA and TFOA (data not shown); this runs counter to a theory that altered patellar alignment causes increase in PF joint stress (e.g., a lateral tilt of the patella leads to increased contact stress on the lateral facet) that contribute to PF joint pain via nociceptive mechanisms. Thus, the fact that patients with coexisting PFOA had worse clinical symptoms does not have specific implications for compartment-specific intervention.

## Conclusions

Clinical symptoms and functional disability in patients with coexisting PFOA were overall stable rather than worsening at the 1-year follow-up. This indicates that patients with coexisting PFOA may be a clinically severe disease subgroup. Additionally, subgroup analysis revealed that patients with isolated lateral PFOA had mild/moderate knee pain, and their VAS scores were likely to improve, whereas those with mixed PFOA exhibited stable to worsening moderate/severe knee pain. This suggests that patients with coexisting PFOA might have heterogeneous clinical outcomes, and presence of mixed PFOA might be an indicator of severe clinical knee OA.

## Additional file

Additional file 1: Table S1. Comparison of baseline characteristics between patients with isolated lateral, isolated medial, and mixed medial and lateral (*n* = 23) PFOA*. (DOCX 38 kb)

## Abbreviations

ANCOVA: Analysis of covariance
BMI: Body mass index
JKOM: Japanese Knee Osteoarthritis Measure
JSN: Joint space narrowing
K/L grade: Kellgren/Lawrence grade
MRI: Magnetic resonance imaging
OA: Osteoarthritis
OARSI: Osteoarthritis Research Society International
PF: Patellofemoral
TF: Tibiofemoral
VAS: Visual analog scale
