# Supplementary material for: Changes in clinical symptoms and functional disability in patients with coexisting patellofemoral and tibiofemoral osteoarthritis: a 1-year prospective cohort study
Source: BMC Musculoskelet Disord. 2017 Mar 24;18:126. doi: 10.1186/s12891-017-1486-4 (PMC5364585; doi:10.1186/s12891-017-1486-4)
Supplement: Additional file 1: Table S1. — Comparison of baseline characteristics between patients with isolated lateral, isolated medial, and mixed medial and lateral (n = 23) PFOA*. (DOCX 38 kb) [file 12891_2017_1486_MOESM1_ESM.docx]

Additional file 1: Table S1. Comparison of baseline characteristics between patients with isolated lateral, isolated medial, and mixed medial and lateral (n = 23) PFOA*

| Variables | Isolated lateral (n = 18) | Isolated medial (n = 4) | Mixed (n = 23) |
| --- | --- | --- | --- |
| Age, years | 75.7 ± 10.2 | 72.5 ± 11.2 | 75.4 ± 7.66 |
| Women, no. (%) | 15 (83.3) | 3 (75) | 15 (65.2) |
| Height, meters | 1.54 ± 0.09 | 1.52 ± 0.07 | 1.54 ± 0.08 |
| Weight, kg | 57.5 ± 9.86 | 64.6 ± 9.68 | 60.4 ± 10.6 |
| Body mass index, kg/m^2^ | 24.1 ± 3.37 | 28.1 ± 3.97 | 25.4 ± 3.85 |
| Tibiofemoral joint K/L grade, no. (%) |  |  |  |
| grade 2 | 16 (88.9) | 2 (50.0) | 5 (21.7) |
| grade 3 | 1 (5.6) | 2 (50.0) | 10 (43.5) |
| grade 4 | 1 (5.6) | 0 (0) | 8 (34.8) |
| Patellofemoral joint K/L grade, no. (%) |  |  |  |
| grade 0 | 0 (0) | 0 (0) | 0 (0) |
| grade 1 | 0 (0) | 0 (0) | 0 (0) |
| grade 2 | 16 (88.9) | 4 (100) | 10 (43.5) |
| grade 3 | 1 (5.6) | 0 (0) | 8 (34.8) |
| grade 4 | 1 (5.6) | 0 (0) | 5 (21.7) |
| Patellar alignment and trochlear morphology |  |  |  |
| Lateral displacement, % | 11.0 ± 4.97 | 8.00 ± 5.29 | 10.0 ± 6.67 |
| Tilting angle, degrees† | 7.37 ± 4.37 | 3.00 ± 0.79 | 3.00 ± 3.23 |
| Sulcus angle, degrees | 135.9 ± 6.33 | 137.0 ± 2.22 | 131.0 ± 6.36 |

PFOA: Patellofemoral osteoarthritis, K/L grade: Kellgren/Lawrence grade.

* Except where otherwise indicated, values are mean ± SD.

† A positive value for tilting angle indicates patellar tilt toward the lateral side and a negative value to the medial side.
